# Clinical Characteristics of Spinal versus General Anaesthesia in Older Patients Undergoing Hip Fracture Repair Surgery in Jordan: A Multicentre Study

**DOI:** 10.3390/jpm13111611

**Published:** 2023-11-16

**Authors:** Lou’i Al-Husinat, Sarah Al Sharie, Mohammad Araydah, Zaid Al Modanat, Mohammed I. A. Ismail, Hadeel B. Heilat, Mohd Said Dawod, Khaled Ahmad Sawaftah, Silvia De Rosa, Denise Battaglini

**Affiliations:** 1 Department of Clinical Sciences, Faculty of Medicine, Yarmouk University, Irbid 21163, Jordan; zaid.modanat@yu.edu.jo (Z.A.M.); hadeel.heilat@yu.edu.jo (H.B.H.); 2 Faculty of Medicine, Yarmouk University, Irbid 21163, Jordan; 3 Department of Internal Medicine, Istishari Hospital, Amman 11942, Jordan; mohaari98@gmail.com; 4 Anesthesiology Department, Mut’ah School of Medicine, Al-Karak 61710, Jordan; 5 Department of Special Surgery, College of Medicine, Mutah University, Al-Karak 61710, Jordan; msd906@gmail.com; 6 Department of General Surgery, Jordan Hospital, Amman 11152, Jordan; khaled267sawaftah@gmail.com; 7 Centre for Medical Sciences—CISMed, University of Trento, 38122 Trento, Italy; derosa.silvia@ymail.com; 8 Anesthesia and Intensive Care, Santa Chiara Regional Hospital, APSS, 38122 Trento, Italy; 9 Anesthesia and Intensive Care, IRCCS Ospedale Policlinico San Martino, 16132 Genova, Italy; battaglini.denise@gmail.com

**Keywords:** hip fracture, spinal anaesthesia, general anaesthesia, femur neck fracture, geriatric

## Abstract

Background: The primary aim of this study was to examine the clinical characteristics and outcomes of older patients who underwent hip fracture repair surgery. The secondary aims were to assess the predictors of the choice of spinal or general anaesthesia and to explore the risk factors for all-cause mortality. Methods: This three-tertiary centres study was conducted at a tertiary care centre in Jordan. Clinical data include previous fracture history; medication details; comorbidities; surgical approach; and postoperative pain management. Results: Overall, 1084 patients who underwent hip fracture repair were included in this study. The mean age of patients was 78 years, and 55.2% were women. Twenty-four were treated with bisphosphonates before the fracture, whereas 30 were in steroid therapy. Overall, 61.8% of patients underwent spinal anaesthesia, whereas 38.2% underwent general anaesthesia. Spinal anaesthesia group had a lower prevalence of cardiovascular accidents (16.3% vs. 22.3%, *p* = 0.014) and Alzheimer’s (3.4% vs. 1.4%, *p* = 0.049) than the general anaesthesia group. In the spinal anaesthesia group, postoperative opioid administration (*p* = 0.025) and postoperative blood transfusion (*p* = 0.011) occurred more frequently than general anaesthesia group. In hospital, 30-day and all-cause mortality were comparable between both groups. Diabetes mellitus (HR = 2.6; 95%CI = 1.5–4.4; *p* = 0.001); cemented hip hemiarthroplasty (HR = 2.4; 95%CI = 1.1–5.1; *p* = 0.025); deep venous thrombosis/pulmonary embolism (HR = 5.0; 95%CI = 1.2–12.9; *p* = 0.001); and readmission within 1 month from surgery (HR = 3.6; 95%CI = 2.0–6.3; *p* < 0.001) were all significant predictors of mortality. Conclusions: This study provides insights into the outcomes and factors associated with different anaesthesia types in hip fracture repair surgery. The anaesthesia type does not affect all-cause mortality in patients undergoing hip fracture repair.

## 1. Introduction

Hip fractures are common injuries among the elderly population and are associated with significant morbidity and mortality [1].

Surgical intervention is often considered the preferred treatment approach for hip fractures in elderly patients, to ensure early mobilization, reduce complications, and improve functional outcomes, and requires a delicate balance between achieving effective pain control and minimizing the risks associated with anaesthesia [2]. The choice between spinal and general anaesthesia for hip fracture surgery in older patients has been an ongoing debate in the past [3].

Spinal and general anaesthesia are two primary techniques commonly employed in hip fracture surgeries. Each method carries its own set of advantages and limitations, making the decision-making process complex and multifaceted [2].

Spinal anaesthesia offers the advantage of targeted pain relief, a lower incidence of respiratory complications, and a reduced risk of confusion or cognitive dysfunction, which can be particularly advantageous in older patients with pre-existing medical conditions [4]. Furthermore, it allows for early postoperative mobilization, promoting faster recovery and shorter hospital stays [4].

General anaesthesia may be considered more versatile and suitable for patients with complex medical histories or those who cannot tolerate spinal anaesthesia [5]. However, older patients undergoing hip fracture surgery under general anaesthesia may experience a higher risk of postoperative delirium, prolonged sedation, and respiratory complications [6]. Moreover, delayed mobilization is possible, leading to a higher incidence of complications such as deep vein thrombosis (DVT) and pressure sores [7]. The decision regarding the anaesthesia technique in older patients undergoing hip fracture surgery must be carefully tailored to health status, pre-existing comorbidities, cognitive function, and preferences [6]. No conclusions can be drawn for longer-term mortality.

The need for a cohort study about geriatric hip fracture patients in Jordan is necessary to gather more recent and region-specific data that can inform targeted interventions and healthcare policies for this vulnerable population. The primary aim of this study was to examine clinical characteristics and outcomes of patients who underwent hip fracture repair surgery during the perioperative period in three tertiary care hospitals in Jordan. The secondary aims were to assess the predictors among comorbidities associated with the choice of spinal or general anaesthesia and to explore the risk factors for all-cause mortality.

## 2. Materials and Methods

### 2.1. Design, Setting, and Participants

This study was conducted across three tertiary care centres and focused on elderly patients who underwent surgery to repair hip fractures. The timeframe of the study spanned from January 2019 to January 2021. The hospitals involved in this research were located in different regions and included the Jordan University Hospital (JUH) in Amman, Jordan, and the King Abdullah University Hospital in Irbid, Jordan, and Ministry of Health hospitals, Amman, Jordan. This study was approved by the Institutional Review Board of Yarmouk University, Irbid, Jordan, reference-2023/133, and follows the principles of Good Clinical Practice and the Declaration of Helsinki [8].

### 2.2. Inclusion and Exclusion Criteria

The inclusion criteria were age 65 years or older, patients diagnosed with any neck femur or hip fracture, patients who underwent hip fracture surgical repair, and signed consent. Patients were excluded if they did not undergo surgery for hip fractures or if their medical notes were not accessible.

### 2.3. Preoperative Care

Preoperative care included providing 500 mL–1 L of normal saline before anaesthesia. Preoperative anticoagulant treatment was subcutaneous calcium heparin 12 h before surgery. Low molecular weight heparin was discontinued 24 h before the procedure. Aspirin and clopidogrel were discontinued 7 and 14 days before the procedure, respectively.

### 2.4. Intraoperative Care

Standard monitoring included continuous electrocardiography, pulse oximetry, and blood pressure measurements. Patients receiving spinal anaesthesia were carefully turned with the fracture side up for performing lumbar puncture using a 22/25-gauge Quincke point needle, positioned midline at the L3–L4 interspace by an experienced physician.

The anaesthetic solution for spinal anaesthesia comprised 10 mg bupivacaine + 25 µg fentanyl. The injection was made over 10 s, needle side opening, and without aspiration of the spinal fluid to avoid paddling, which may render the spinal anaesthesia bilateral. The lateral position was maintained for 15 min, and the patients were then reinstalled in dorsal decubitus or left inside decubitus according to the intervention. The level of sensory blockade and intensity of motor blockade were bilaterally evaluated 15 min after spinal anaesthesia using the modified Bromage scale.

Patients undergoing general anaesthesia received continuous intravenous 100 mg propofol + 100 µg fentanyl. Tracheal intubation was performed, and rocuronium was used as a muscle relaxant. Anaesthesia was maintained using sevoflurane (MAC = 2%), at the discretion of the attending anaesthesiologist.

### 2.5. Postoperative Care

Postoperative analgesia was initiated 30 min before the predictable end of surgery. Intravenous administration of 5 mg morphine sulphate was performed to relief pain. All patients received DVT prophylaxis with 40 mg apixaban 14 days following surgery or extended to 35 days in patients at high risk for DVT. The occurrence of postoperative complications within the first postoperative day was recorded.

### 2.6. Data Collection and Definitions

Clinical data were collected from electronic medical records, including basic demographics (age, sex, smoking status, comorbidities, medications, and time of surgery), preoperative information (preoperative analgesia and antibiotics, haemoglobin level), hip fracture (open or closed: subtrochanteric, intertrochanteric, and femoral neck fractures), intraoperative information (type of anaesthesia, fixation type, cementation status, and type of hemiarthroplasty) and post-operative information (ICU admission, haemoglobin level, blood transfusion, analgesia, readmissions to hospital, in-hospital mortality, 30-day mortality and all-cause mortality). All-cause mortality was defined as death from any cause among individuals who have undergone hip fracture surgery from the post-operative period to last follow-up at 12 months.

### 2.7. Statistical Analysis

A sample size calculation was not performed, given the retrospective nature of this study. All consecutive patients undergoing hip fracture repair over two years were included. Normality of variables was assessed using histograms or quantile–quantile plots in addition to the Kolmogorov–Smirnov and Shapiro–Wilk tests. The means with standard deviation were calculated for the normally distributed continuous variables. Median with Interquartile range (IQR) was used to describe non-normal continuous variables, whereas frequencies and percentages were used to describe categorical variables. Continuous non-normally distributed variables were compared using the Mann–Whitney test, whereas categorical variables were compared using chi-square test. Multivariable logistic regression analysis with Odds ratio (OR) was performed to explore the association between patients’ comorbidities and the type of anaesthesia. Variables with a *p*-value less than 0.2 on the Chi-Square, *t* test, and Mann–Whitney teste were included in this analysis. All the variables included were subjected to univariable logistic regression to assess potential associations with mortality. Only significant variables on univariable logistic regression (diabetes mellitus, cemented hip arthroplasty, DVT/pulmonary embolism (PE), and readmission within one month) were included in Cox hazard regression analysis. All data were analysed using Stata version 17 software (StataCorp. 2021. Stata: Release 17. Statistical Software. College Station, TX, USA: StataCorp LLC.). The statistical significance was set at a 2-sided *p* < 0.05.

## 3. Results

### 3.1. Patients and Treatments

A total of 1084 older patients who underwent hip fracture repair were included in this retrospective study. The mean age of patients was 78 years ± 7.3, with 598 (55.2%) women. Twenty-four (2.2%) had been treated with bisphosphonates before fracture, whereas 30 (2.8%) were in steroid therapy. Patients’ demographics and clinical characteristics are described in Table 1. Overall, 61.8% (670) of patients underwent spinal anaesthesia, whereas 38.2% (414) had general anaesthesia. The median age of patients who received spinal anaesthesia was significantly lower than those who received general anaesthesia (78 vs. 77 years, *p* = 0.0443), but with a significantly lower prevalence of cardiovascular accidents (16.3% vs. 22.3%, *p* = 0.014) and Alzheimer (3.4% vs. 1.4%, *p* = 0.049). Compared to patients undergoing spinal anaesthesia, those undergoing general anaesthesia were treated less frequently with anticoagulants other than aspirin and clopidogrel (8.2% vs. 4.8%, *p* = 0.033). The median days from admission to surgery were significantly lower in patients receiving spinal than general anaesthesia (2 vs. 3 days, *p* < 0.001). Other variables, including sex, smoking status, other comorbidities, and other medications used, were comparable between both groups, as shown in Table 1.

### 3.2. Preoperative Care

Table 2 includes preoperative variables of patients who underwent surgical hip fracture repair. Patients who underwent surgery under spinal anaesthesia received cefuroxime or cefazolin significantly less than those who received general anaesthesia (78.3% vs. 87.6%, *p* = 0.001). Furthermore, those who had surgery under spinal anaesthesia received significantly more frequent cefuroxime than general anaesthesia (18.3% vs. 5.0%, *p* < 0.001). Other variables, including ICU admission, types of analgesia used, and other antibiotics, were comparable between groups.

### 3.3. Intraoperative Care

Table 3 includes intraoperative variables and characteristics of hip fractures of patients undergoing hip fracture repair. In both spinal and general anaesthesia groups, the most common fracture types were unstable intertrochanteric fractures (46.1% spinal, 44.9% general) and unstable femoral neck fractures (29.6% spinal, 25.6% general). Furthermore, stable femoral neck fractures were significantly less common in patients who received spinal anaesthesia than general anaesthesia (3.1% vs. 6.8%, *p* = 0.005). Other types of hip fractures were comparable between both groups.

In both groups, the most common fixation type to be used were intramedullary nailing (62.4% spinal and 65.0% general anaesthesia group) and hip hemiarthroplasty (30.3% spinal and 27.8% general anaesthesia group). Additionally, hip fracture fixation through cannulated screws was notably lower spinal anaesthesia compared to general anaesthesia group (0.2% vs. 1.5%, *p* = 0.009). Other fixation types were comparable between both groups.

Patients who underwent spinal anaesthesia had a higher percentage of cemented hip arthroplasty than those who received general anaesthesia (81.3% vs. 61.5%, *p* < 0.001). Additionally, unipolar or bipolar hip hemiarthroplasty were comparable between groups (unipolar = 15.3% vs. 9.6%, *p* = 0.103; bipolar = 84.7% vs. 90.4%, *p* = 0.840).

### 3.4. Postoperative Care

Table 4 shows the postoperative outcomes of patients undergoing hip fracture repair. Compared to the general anaesthesia group, the spinal anaesthesia group had a significantly higher prevalence of postoperative opioid administration (63.1% spinal vs. 56.3% general, *p* = 0.025) and postoperative blood transfusion (38.8% spinal vs. 31.2% general, *p* = 0.011).

### 3.5. Predictors of Type of Anaesthesia

**Figure 1:**
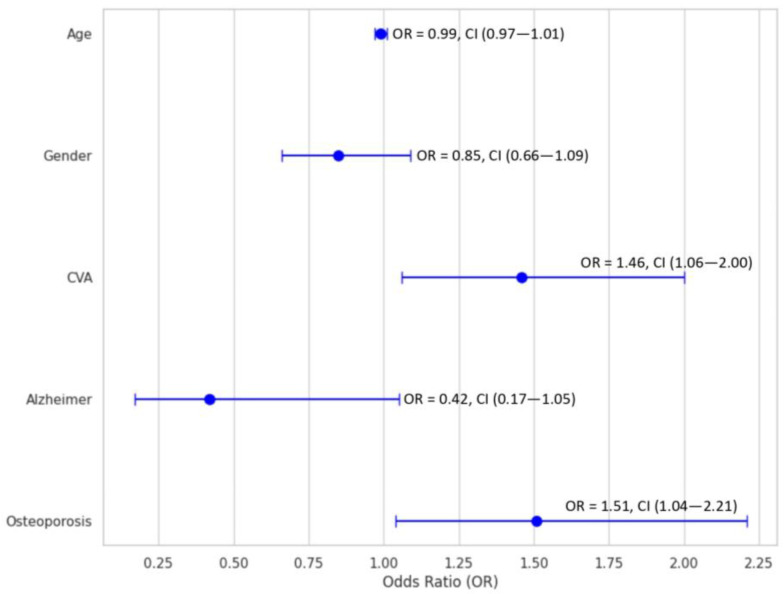
Adjusted multivariable analysis of various predictors by the type of anaesthesia (spinal vs. general). CVA: Cerebral Vascular Accident.

Figure 1 shows the multivariable logistic regression analysis exploring the association between patients’ comorbidities and the type of anaesthesia. Patients with a history of CVAs (OR = 1.5, 95%CI = 1.1–2.0, *p* = 0.019) and osteoporosis (OR = 1.5, 95%CI = 1.0–2.2, *p* = 0.032) were more likely to receive spinal than general anaesthesia.

### 3.6. Mortality and Risk Factors for Mortality

**Figure 2:**
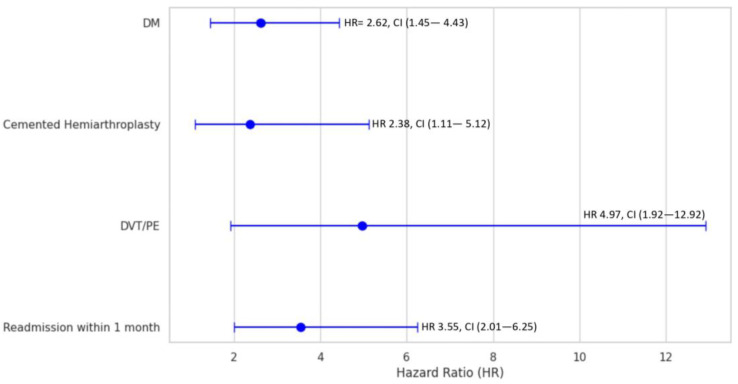
Multivariable cox hazard regression analysis of various variables in patients undergoing hip fracture repair. DM: Diabetes Mellitus, DVT: Deep Vein Thrombosis, PE: Pulmonary Embolism.

As shown in Figure 2, patients with diabetes mellitus had approximately two and half-fold higher risk of mortality (HR = 2.6, 95%CI = 1.5–4.4, *p* = 0.001). Moreover, those who underwent cemented hip hemiarthroplasty had a two-fold higher risk of mortality (HR = 2.4, 95%CI = 1.1–5.1, *p* = 0.025). DVT/PE was associated with an approximately five-fold higher mortality risk (HR = 5.0, 95%CI = 1.9–12.9, *p* = 0.001). Three and half-fold higher mortality risk was observed in those who had readmission within 1 month of surgery (HR = 3.6, 95%CI = 2.0–6.3, *p* < 0.001).

At 30 days, mortality did not significantly differ between spinal and general anaesthesia groups (*p* = 0.904) (Table 4). All-cause overall mortality did not significantly differ between groups. (*p* = 0.466).

## 4. Discussion

The present study examined the clinical characteristics and outcomes of patients who underwent hip fracture repair surgery in three tertiary care hospitals in Jordan.

The main strength of this study is that it provides detailed and essential information about patients with surgical management of hip fractures, reporting perioperative outcomes that may help improve the delivered healthcare. These data are important because they report perioperative outcomes that can help improve the health care provided. To the best of our knowledge, only two single-centre studies and one multicentre epidemiological study previously investigated this topic in Jordan, but no one was specific on anaesthetic management in older patients [9,10,11].

Our data showed that anaesthetic management is more frequent in general anaesthesia than spinal anaesthesia, particularly for unstable intertrochanteric and femoral neck fractures. According to the literature, general anaesthesia is more frequently used in patients undergoing surgical repair of hip fractures than spinal anaesthesia [12,13,14,15,16,17]. For instance, Nawi et al. found that a more significant % of elderly patients who underwent hip fracture repair (72.7%) received general anaesthesia. Spinal anaesthesia was administered to 27.3% of the patients [13].

In our study, the average age of patients who received spinal anaesthesia was higher than those who received general anaesthesia (78.4 vs. 77.6 years), which may have influenced the anaesthetic choice.

The present study found that the most common fracture types were unstable intertrochanteric fractures and unstable femoral neck fractures. This finding is consistent with the literature [18]. Intramedullary nailing (63.4%) and hip hemiarthroplasty (29.4%) were the most common fixation types used in the present study, as observed in previous studies [2,19].

Our study found that patients who received general anaesthesia tended to stay at the hospital longer than those who received spinal anaesthesia. Neuman et al. found that regional anaesthesia was associated with a reduction in hospital length of stay by half a day. Nawi et al. found that patients who received general anaesthesia exhibited an increased length of stay in the hospital (OR = 1.3, 95%CI = 1.0–1.5; *p* = 0.02) [13]. This finding could be related to the demographic trend, which indicates that patients who receive regional anaesthesia generally tend to be older and have more complex medical conditions than those who choose general anaesthesia [20].

Patients with a postoperative higher occurrence of blood transfusions and more extended hospital stay were more likely to have received spinal anaesthesia. However, these data contrasted with recent meta-analysis showing a shorter hospital stay and a reduced need for blood transfusions in patients undergoing hip fracture surgery under spinal anaesthesia [21]. Compelling evidence indicates that patients undergoing hip fracture surgery experience higher intra-operative arterial blood pressure levels when subjected to spinal anaesthesia, thus increasing the rate of intra-operative blood loss [22]. In contrast to our findings, Morgan et al. found that receiving spinal anaesthesia was associated with a reduced likelihood of requiring blood transfusion as compared to general anaesthesia (OR = 0.8, 95%CI = 0.8–0.9, *p* = 0.003) [23] Lončarić-Katušin et al. showed that the type of anaesthesia does not affect postoperative mortality [24]. Although strategies to mitigate bleeding have been implemented, patients undergoing spinal anaesthesia had a high percentage of home therapy with aspirin, clopidogrel, or other anticoagulant agents associated with a preoperative haemoglobin level of less than 10 times higher in the general anaesthesia group.

At multivariable logistic regression analysis, patients with a medical history of CVAs or osteoporosis exhibited a 150% increased likelihood of receiving spinal anaesthesia. Nawi et al. found that patients in residential care facilities were likelier to have hip fracture surgery under general anaesthesia than spinal anaesthesia (OR = 2.9, 95%CI = 1.1–7.4; *p* = 0.03) [12]. Furthermore, dementia was higher in patients living in residential care facilities compared to community dwellers [12]. Over half of the residents in the residential care facilities require mobility aids or are bedbound [12]. Theou et al. found that approximately 50% of patients residing in residential care facilities develop frailty [25]. The consideration of anaesthesia choice may have been influenced by physical dependence and frailty, as these conditions can potentially heighten the technical complexities involved in administering regional anaesthesia.

Regarding mortality outcomes, there were no significant differences in in-hospital, 30-day, and all-cause mortality rates between the spinal and general anaesthesia groups. However, certain factors significantly predicted higher mortality after hip fracture repair in our study, including the presence of diabetes mellitus, the use of a cemented hip, and the occurrence of postoperative DVT/PE complications. Patients who experienced readmission within one month after the hip fracture repair were also found to be at a higher risk of mortality. The conclusions drawn regarding mortality were consistent with the current evidence. Zuo et al. conducted a meta-analysis of seven randomized controlled trials, revealing that there was no significant difference in 30-day mortality between elderly patients who underwent surgical repair of hip fractures with spinal anaesthesia compared to those who had general anaesthesia (relative risk (RR) = 1, 95%CI = 0.9–1.0, *p* = 0.48) [26]. Furthermore, in a more recent systematic review and meta-analysis by Kunutsor et al. comparing spinal anaesthesia to general anaesthesia, the RR for mortality was 0.6, 95%CI = 0.2–1.4 in-hospital, 1.1, 95%CI = 0.5–2.2 at 30 days, and 1.1, 95%CI = 0.55–2.12 at 90 days [27].

Although previous studies have documented that spinal anaesthesia is associated with improved outcomes and has been shown to decrease the mortality rate and pulmonary complications by 30% [14,28], Neuman et al. showed no difference in survival and recovery of ambulation at 60 days between spinal and general patients with a hip fracture. The incidence of postoperative delirium was similar between groups [29].

Numerous studies have compared postoperative complication rates between spinal and general anaesthesia. The rates of urinary tract infections were reduced in patients with general anaesthesia compared to those with spinal anaesthesia [28]. Whiting et al. demonstrated that superficial wound infection (OR = 2.0, 95%CI = 1.0–3.4, *p* = 0.04) and coma (OR = 3.4, 95%CI = 1.0–11.0, *p* = 0.041) were more likely to occur in patients with spinal anaesthesia [12]. In our study, the only complication available to be compared across both groups was DVT/PE, but without statistically significant differences between the two groups.

Finally, in our study, the presence of diabetes mellitus, the use of cemented hip arthroplasty, the occurrence of postoperative DVT/PE, and readmission within one month were all significant predictors for higher mortality after hip fracture repair. A meta-analysis by Shen et al. determined a higher risk of mortality among individuals with diabetes mellitus compared to those without after one year [30]. The RR was 1.2, 95%CI = 1.1–1.4. However, statistical heterogeneity with an I2  =  62% underscored the meaningfulness of this finding. Garland et al. found that individuals who underwent cemented total hip arthroplasty exhibited a higher likelihood of mortality within the initial 14 days in comparison to the control group (HR = 1.3, 95%CI = 1.1–1.4) [31]. The combined mortality rates at 1 month, 3 months, and 6 months were significantly elevated in patients with post-operative PE (16.1%, 23.0%, and 28.6%, respectively), as opposed to patients without this complication, who had lower rates (3.3%, 6.7%, and 10.2%, respectively) [32].

A study by Moldovan et al. suggests that the type of osteosynthesis performed has significant implications on the surgical trauma experienced by elderly patients with hip fractures, with the duration of surgery and the length of hospital stay being crucial predictive factors. These findings can guide clinical decision-making to minimize the impact of surgical trauma on this vulnerable patient group [33].

This study has several limitations that need to be addressed. First, although race, multiparity, socioeconomic status, menopause, addictions, and family history are all well-known risk factors for osteoporosis, these data have not been reported in patient records and analysis. Furthermore, we have no data relating to the preoperative condition of vitamin-D deficiency or intake of hormonal drugs that could have influenced the incidence of fractures, mainly present in females. Secondly, we have no data on the number of patients managed conservatively nor the reasons for avoiding surgery. Third, for patients who were instead considered for analysis because they were managed surgically, we have no data on the time between admission and surgery, the reason for the delay, the type of surgery, and postoperative complications. Fourth, the information in the medical record regarding patients’ preferences for the type of anaesthesia was not documented.

Furthermore, the reason for the specific anaesthesia approach chosen was not reported. This absence of information could raise questions about how decisions have been made. Fifth, we need to determine what premedication was used to position patients for spinal anaesthesia, the level of neuraxial block achieved, or what doses were administered. Sixth, the results of this study may have been influenced by the lack of information on the patients’ pre-existing coagulopathy, producing a bias or element of uncertainty, requiring caution in interpreting these results. Future research using in-depth patient coagulopathy data may better understand the findings. Further larger RCTs are needed and should consider these elements when designing the clinical trial.

## 5. Conclusions

The findings of our study present a nuanced view of anaesthesia choice in hip fracture repair surgeries among elderly patients. Our data suggest that while spinal anaesthesia is associated with shorter wait times for surgery and a lower prevalence of cardiovascular incidents, general anaesthesia is utilized less frequently with certain preoperative medications. Moreover, the type of anaesthesia does not significantly influence the 30-day mortality rate.

In light of these results, we recommend spinal anaesthesia as the preferred option for hip fracture surgeries in older adults, given its association with expedited surgery and lower cardiovascular complication rates. However, the choice should still be tailored to the individual patient’s medical history, medication use, and specific health considerations.

By adopting a more patient-centric approach that considers both the benefits and risks of anaesthesia types, we aim to optimize outcomes and enhance the quality of care for our elderly patients undergoing hip fracture repair.

## Figures and Tables

**Table 1:** Demographics and clinical characteristics of patients undergoing hip fracture repair.

	Total (n = 1084)	Spinal Anaesthesia (n = 670)	General Anaesthesia (n = 414)	*p*-Value
Age (years), median (IQR)	78 (83–73)	78 (83–73)	77 (82–72)	0.0443
Sex, n (%)				0.165
Male	485 (44.78)	289 (43.13)	196 (47.46)	
Female	598 (55.22)	381 (56.87)	217 (52.54)	
Smoking Status, n (%)				0.310
Smoker	285 (26.29)	169 (25.22)	116 (28.02)	
Non-Smoker	799 (73.71)	510 (74.78)	298 (71.98)	
Comorbidities, n (%)				
Diabetes mellitus	534 (49.26)	325 (48.51)	209 (50.48)	0.527
Hypertension	743 (68.54)	460 (68.66)	283 (68.36)	0.918
Cardiovascular	335 (30.9)	216 (32.24)	119 (28.74)	0.226
CVA	201 (18.56)	109 (16.27)	92 (22.28)	0.014
Pulmonary diseases	47 (4.34)	30 (4.48)	17 (4.11)	0.771
Thyroid diseases	46 (4.24)	27 (4.03)	19 (4.59)	0.657
Renal failure	88 (8.12)	53 (7.91)	35 (8.45)	0.750
Parkinson	36 (3.32)	24 (3.58)	12 (2.90)	0.542
Dementia	11 (1.01)	8 (1.19)	3 (0.72)	0.454
Alzheimer	29 (2.68)	23 (3.43)	6 (1.41)	0.049
Osteoporosis	126 (11.62)	68 (10.15)	58 (14.01%)	0.054
Medications Used, n (%)				
Aspirin	492 (45.39)	296 (44.18)	196 (47.34)	0.309
Clopidogrel	86 (7.93)	50 (7.46)	36 (8.7)	0.466
Other anticoagulants	75 (6.92)	55 (8.21)	20 (4.83)	0.033
Steroids	30 (2.77)	16 (2.39)	14 (3.38)	0.333
Bisphosphonates	24 (2.21)	11 (1.64)	13 (3.14)	0.103
Days from admission to surgery; median (IQR)	2 (4–1)	2 (4–1)	3 (4–2)	<0.001
Days from admission to surgery; n (%)				<0.001
Zero (same day as the operation)	8 (0.74)	3 (0.45)	5 (1.21)	
One	381 (35.15)	290 (43.28)	91 (21.98)	
Two	236 (21.77)	134 (20)	102 (24.64)	
Three or more	459 (42.34)	243 (36.27)	216 (52.17)	

CVA: Cerebral Vascular Accident. Data are presented as numbers (percentages %) or median (interquartile range IQR), as appropriate.

**Table 2:** Preoperative variables of patients undergoing hip fracture repair.

	Total	Spinal Anaesthesia (n = 670)	General Anaesthesia (n = 414)	*p*-Value
Haemoglobin (g/dl), mean (SD)	11.98 ± 1.85	11.95 ± 1.80	12.02 ± 1.93	0.5467
ICU admission, n (%)	28 (2.58)	14 (2.09)	14 (3.38)	0.193
Analgesia type, n (%)				
Paracetamol	1070 (98.71)	660 (98.51)	410 (99.03)	0.456
NSAIDs	9 (0.83)	7 (1.04)	2 (0.49)	0.332
Opioids	445 (41.05)	281 (41.94)	164 (39.61)	0.449
Antibiotics used, n (%)	1041 (96.03)	645 (96.27)	396 (95.65)	0.613
Type of Antibiotic, n (%)				
Cefuroxime or cefazolin	852 (81.84)	505 (78.29)	347 (87.63)	0.001
Vancomycin	23 (2.21)	10 (1.55)	13 (3.28)	0.067
Cefuroxime and vancomycin	138 (13.26)	118 (18.29)	20 (5.05)	<0.001
Ceftriaxone	23 (2.21)	10 (1.55)	13 (3.28)	0.067
Other	5 (0.48)	2 (0.31)	3 (0.76)	0.314

ICU: Intensive Care Unit; NSAIDs: Non-Steroidal Anti-Inflammatory Drug. Data are presented as numbers (percentages %) or mean (standard deviation SD) as appropriate.

**Table 3:** Intraoperative variables of patients undergoing hip fracture repair.

	Total	Spinal Anaesthesia (n = 670)	General Anaesthesia (n = 414)	*p*-Value
Fracture type n (%)				
Stable femoral neck	49 (4.52%)	21 (3.13%)	28 (6.76%)	0.005
Unstable femoral neck	305 (28.14%)	198 (29.55%)	107 (25.85%)	0.187
Stable intertrochanteric	223 (20.57%)	134 (20%)	89 (21.5%)	0.553
Unstable intertrochanteric	495 (45.66%)	309 (46.12%)	186 (44.93%)	0.702
Subtrochanteric	12 (1.11%)	8 (1.19%)	4 (0.97%)	0.728
Fixation type n (%)				
DHS	70 (6.46%)	48 (7.16%)	22 (5.31%)	0.229
IMN	687 (63.38%)	418 (62.39%)	269 (64.98%)	0.390
Hip Hemiarthroplasty	318 (29.34%)	203 (30.3%)	115 (27.78%)	0.376
THR	2 (0.18%)	0 (0%)	2 (0.48%)	0.072
Cannulated screws	7 (0.65%)	1 (0.15%)	6 (1.45%)	0.009
Cement status, n (%)				<0.001
Cemented	237 (74.06%)	165 (81.28%)	72 (61.54%)	
Cementless	83 (26.94%)	38 (18.72%)	45 (38.46%)	
HEMI type, n (%)				
Unipolar	42 (13.21%)	31 (15.27%)	11 (9.57%)	0.103
Bipolar	276 (86.79%)	172 (84.73%)	104 (90.43%)	0.840

DHS: Dynamic Hip Screw, HEMI: Hemiarthroplasty, IMN: Intramedullary Nailing, THR: Total Hip Replacement. Data are presented as numbers (percentages %).

**Table 4:** Postoperative variables of patients undergoing hip fracture repair.

	Total	Spinal Anaesthesia (n = 670)	General Anaesthesia (n = 414)	*p*-Value
ICU admission, n (%)	117 (10.79%)	78 (11.64%)	39 (9.42%)	0.252
Analgesia used, n (%)				
Paracetamol	1075 (99.17%)	665 (99.25%)	410 (99.03%)	0.698
NSAIDs	12 (1.11%)	6 (0.9%)	6 (1.45%)	0.397
Opioids	656 (60.52%)	423 (63.13%)	233 (56.28%)	0.025
Haemoglobin (g/dL), median (IQR)	10.30 (11.40–9.20)	10.30 (11.40–9.20)	10.40 (11.40–9.30)	0.5919
Blood transfusion, n (%)	389 (35.89%)	260 (38.81%)	129 (31.16%)	0.011
DVT/PE, n (%)	25 (2.31%)	16 (2.39%)	9 (2.18%)	0.824
Duration of hospital stay (days), median (IQR)	6 (9–5)	6 (8–4)	6 (9–5)	0.0023
Readmission at 1 month, n (%)	113 (10.42%)	71 (10.6%)	42 (10.14%)	0.813
Cause of readmission, n (%)				0.070
Medical Issue	86 (76.11%)	58 (81.69%)	28 (66.67%)	0.262
Fracture/Operation Related	27 (23.89%)	13 (18.31%)	14 (33.33%)	0.139
Revision for same operation, n (%)	29 (2.68%)	14 (2.09%)	15 (3.62%)	0.128

ICU: Intensive Care Unit, DVT: Deep Vein Thrombosis, NSAID: Non-Steroidal Anti-Inflammatory Drug, PE: Pulmonary Embolism. Data are presented as numbers (percentages %) or median (interquartile range IQR) as appropriate.

## Data Availability

The data presented in this study are available on request from the corresponding author.
